# Targeting the Hepatocyte Growth Factor and c-Met Signaling Axis in Bone Metastases

**DOI:** 10.3390/ijms20020384

**Published:** 2019-01-17

**Authors:** Young Mi Whang, Seung Pil Jung, Meyoung-Kon Kim, In Ho Chang, Serk In Park

**Affiliations:** 1 Department of Urology, Chung-Ang University College of Medicine, Seoul 06973, Korea; ymwhang@cau.ac.kr; 2 Division of Breast and Endocrine Surgery, Department of Surgery, Korea University Anam Hospital, Seoul 02841, Korea; jungspil@korea.ac.kr; 3 Department of Biochemistry and Molecular Biology, Korea University College of Medicine, Seoul 02841, Korea; jerrykim@korea.ac.kr; 4 Department of Medicine, Vanderbilt University School of Medicine, Nashville, TN 37232, USA

**Keywords:** bone, metastasis, microenvironment, osteoblasts, osteoclasts, bone marrow, c-Met and hepatocyte growth factor

## Abstract

Bone metastasis is the terminal stage disease of prostate, breast, renal, and lung cancers, and currently no therapeutic approach effectively cures or prevents its progression to bone metastasis. One of the hurdles to the development of new drugs for bone metastasis is the complexity and heterogeneity of the cellular components in the metastatic bone microenvironment. For example, bone cells, including osteoblasts, osteoclasts, and osteocytes, and the bone marrow cells of diverse hematopoietic lineages interact with each other via numerous cytokines and receptors. c-Met tyrosine kinase receptor and its sole ligand hepatocyte growth factor (HGF) are enriched in the bone microenvironment, and their expression correlates with the progression of bone metastasis. However, no drugs or antibodies targeting the c-Met/HGF signaling axis are currently available in bone metastatic patients. This significant discrepancy should be overcome by further investigation of the roles and regulation of c-Met and HGF in the metastatic bone microenvironment. This review paper summarizes the key findings of c-Met and HGF in the development of novel therapeutic approaches for bone metastasis.

## 1. Introduction

Bone metastasis is uniquely positioned in cancer research because of its diverse cellular populations comprising the metastatic microenvironment. For example, cells in the skeletal system, such as osteoblasts, osteoclasts, and osteocytes, as well as numerous subpopulations, including hematopoietic cells, in the bone marrow all play distinct roles in the progression of bone metastasis [1,2]. Bone metastasis is a major clinical problem for many types of cancers, particularly prostate, breast, lung, and kidney cancers. Bone metastasis is considered a terminal stage disease, and often results in extensive morbidities, such as severe bone pain, spinal cord compression, pathologic fracture, immobility, and ultimately death [3]. Currently, no drugs are available for curing or preventing bone metastasis. Anti-resorptives such as bisphosphonates and denosumab are used to delay or prevent the skeletal-related events (SREs), yet have not been proven to show significant overall survival benefits [4]. This conflict between important unmet clinical needs and the absence of effective therapeutic approaches is mostly due to the complexity in the signaling pathways among the diverse stromal cell types in the metastatic bone microenvironment [5]. For example, the transforming growth factor (TGF)-beta/bone morphogenetic protein (BMP) signaling pathway is the most comprehensively investigated mechanism of cancer-induced bone diseases [6,7]. In addition, the Wnt/beta-Catenin signaling pathway is frequently dysregulated in the bone lesions of multiple myeloma, prostate, and breast cancer metastasis [6,8]. Inhibitors of Wnt antagonists, such as anti-Sclerostin and anti-Dickkopf-related protein 1 (DKK1), have been investigated in the context of bone metastasis. Tyrosine kinases, such as Src family kinases, platelet-derived growth factor receptor (PDGFR), and c-Kit, have previously been highlighted as promising therapeutic targets in cancer-induced osteolysis and metastatic tumor growth in bone [9,10,11,12]. Inhibitors of chemokines and chemokine receptors, such as anti-C-C chemokine ligand 2 antibodies, have also been tested for prostate cancer bone metastasis [13,14]. However, none of these drugs or antibodies resulted in significant benefits during clinical trials in patients with bone metastasis, suggesting that the signaling pathways in the metastatic bone microenvironment are extremely heterogeneous and complex and also that no single target can effectively stop progression of the disease. Accordingly, more extensive delineation of growth factors and their related signaling pathways specific to the metastatic bone microenvironment must be clarified to develop more effective therapeutics for bone metastasis. Recent publications have highlighted hepatocyte growth factor (HGF) and its cognate receptor c-Met as promising therapeutic targets of metastatic tumor growth and osteolysis of bone metastasis. This review will summarize the new findings on the roles of hepatocyte growth factor (HGF) and c-Met receptor tyrosine kinase in the progression of bone metastasis.

## 2. Biology of c-Met and HGF

HGF, also known as Scatter Factor (SF), is a soluble cytokine and belongs to the plasminogen-related growth factor family. HGF/SF was identified by two groups that independently characterized a mitogenic factor for hepatocytes (named HGF) and a fibroblast-derived epithelial cell mobility factor (named SF), respectively, and later, the two factors were identified to be the same [15]. Proteolytic regulation is an important characteristic of HGF. Single chain pro-form HGF is produced and subsequently activated by proteolytic cleavage into disulfide-bonded α- and β-chain heterodimers by serine proteinases such as urokinase plasminogen activator (uPA); tissue-type plasminogen activator (tPA); coagulation factors X, XI, and XII; and a homologue of factor XII. HGF has a strong binding affinity with heparan sulfate proteoglycans abundant in the extracellular matrix, which explains the limited diffusion of HGF throughout the tissue.

c-Met is encoded by the *MET* gene that was cloned and identified as a proto-oncogene by George Vande Woude at the U.S. National Cancer Institute in 1984 [16]. c-Met is a receptor tyrosine kinase, and HGF is the sole ligand for c-Met. Since its discovery, the c-Met receptor has been extensively investigated for its roles in cellular functions and tumor progression, and thus ample review papers are available to read [17,18]. Therefore, only a summary on the c-Met structure and downstream signaling will be briefly covered here. c-Met is a single-pass disulfide-linked 50kDa α- and 140kDa β-subunit heterodimer. The extracellular compartment of c-Met has three domains, including semaphorin, PSI (plexins, semaphorins, and integrins), and IPT (immunoglobulin-plexin-transcription) domains. The intracellular compartment contains a kinase domain and a multifunctional docking site. c-Met activation by ligand binding leads to the phosphorylation of Y1234 and Y1235 in the kinase domain. Subsequently, Y1349 and Y1356 in the multifunctional docking site become phosphorylated, followed by the recruitment of multiple adaptor proteins, such as growth factor receptor-bound protein (Grb) 2; Grb 2-associated binding protein (Gab) 1; Src homology-2-containing (SHC); v-crk sarcoma virus CT10 oncogene homolog (CRK); and CRK like (CRKL), as well as effector molecules such as phosphatidylinositol 3-kinase (PI3K), phospholipase C (PLC) γ and Src, Src homology domain-containing 5′ inositol phosphatase (SHIP)-2, and the transcription factor signal transducer and activator of transcription (STAT)-3. In particular, Gab 1 is a multi-adaptor protein that serves binding sites for numerous downstream adaptors, further diversifying the intracellular signaling pathways. Intracellular downstream signaling pathways of c-Met include Akt/PKB (protein kinase B) regulating cell survival and growth; Src/FAK (focal adhesion kinase) regulating mobility and invasion; JNK (c-Jun *N*-terminal Kinase) regulating transformation; Ras/Raf regulating cell proliferation and cell cycle progression; and PIP3 (phosphatidyl inositol 3,4,5-triphosphate)/PKC (protein kinase C), which works to negatively regulate c-Met signaling, etc.

## 3. c-MET/HGF Signaling in Cancer Progression

c-Met was first cloned using chemically transformed human osteosarcoma cells and was identified as an oncogene as mentioned above [18]. Indeed, the activation of c-Met is frequently observed in many types of cancers. Mechanisms of c-Met activation include gain-of-function mutation, gene amplification, and constitutive over-production of HGF in the microenvironment, and therefore, c-Met can be activated in both ligand-dependent and -independent manners. c-Met can be indirectly activated by growth factors other than HGF, including insulin-like growth factor (IGF)-1, epidermal growth factor (EGF), etc. Varkaris et al. demonstrated that IGF-1 binding to its IGF receptor (IGFR) trans-activates c-Met ligand-independently via Src phosphorylation in prostate cancer tumor cells [19]. Lee et al. demonstrated that a similar mechanism (i.e., IGF-dependent trans-activation of c-Met) exists in osteoblasts [20]. Furthermore, Breindel et al. demonstrated that epidermal growth factor (EGF) trans-activates c-Met via MAPK [21].

Genetic and protein expression analyses of patient tissue samples demonstrated that c-Met activation is closely associated with tumor progression and metastasis in breast and prostate cancers, with a higher expression in the metastatic lesions compared to the primary lesions [22,23,24,25]. c-Met is predominantly expressed in epithelial cells (including carcinoma cells), hepatocytes, and endothelial cells, and HGF expression is limited by mesenchymal cells, such as fibroblasts and muscle cells. c-Met and HGF are essential to the development of the liver and the cardio-vascular system, and thus *Met-* or *Hgf*-knockout mice are both embryonic lethal [26,27,28]. The pro-tumorigenic role of c-Met can function through various downstream signaling pathways, such as Ras/Raf/MAPK, PI3K/Akt, and Wnt/β-catenin, among many others, resulting in multiple biological effects, such as cell proliferation, cell motility, invasion, metastasis, evasion of apoptosis, epithelial-to-mesenchymal transition, and angiogenesis. 

## 4. c-MET/HGF Signaling in Bone Metastasis

One of the earliest and key findings supporting the role of c-Met and HGF in bone metastasis was reported by the Vande Woude group, who demonstrated that prostate cancer bone metastasis patient samples showed significantly increased c-Met protein expression in the metastatic lesions, and also that c-Met expression inversely correlated with androgen receptor expression [29]. The authors obtained and analyzed 90 prostatectomy surgical samples, and discovered that Met is expressed in half of the primary cancers, whereas Met expression is expressed in all metastases. In addition, Met expression inversely correlated with the expression of the androgen receptor, indicating that Met expression increases during the disease progression. Given that HGF is a stromal factor whose receptor, c-Met, is expressed in carcinoma cells, the c-Met/HGF axis was investigated in the context of tumor-stromal interactions. The study from Grano et al. was the first important report describing the expression and the roles of HGF and c-Met in osteoblasts and osteoclasts. The authors demonstrated that human osteoclasts and osteoblasts both express c-Met and that HGF stimulates both cell types. The authors also demonstrated that c-Met activation increases the intracellular calcium concentration and Src phosphorylation in osteoclasts, whereas c-Met activation in osteoblasts induced cell cycle progression, collectively suggesting that HGF is a coupling factor for osteoclasts and osteoblasts [30]. In line with this important finding, we and others have demonstrated that c-Met and HGF play important roles among three compartments in the metastatic bone microenvironments, i.e., tumor cells, osteoclasts, and osteoblasts [20,31,32]. According to the advent of novel tyrosine kinase inhibitors (TKIs), many studies regarding the role of c-Met/HGF in bone metastasis originated from the pre-clinical and clinical studies using novel c-Met TKIs.

Most recently, Smith et al. demonstrated that a novel c-Met and VEGFR dual TKI (cabozantinib) resulted in a partial or complete resolution of bone lesions measured by Tc-99m bone scans and also the improvement of cancer-induced bone pain [33,34]. Briefly, in a Phase 2 nonrandomized expansion study in castration resistance prostate cancer, the patients (*n* = 144) received cabozantinib 100 mg (*n* = 93) or 40 mg (*n* = 51) daily from the start until disease progression or unacceptable toxicity. Cabozantinib treatment resulted in pain relief (57% of patients) measured by a reduction or discontinuation of narcotic analgesics, as well as improvements in bone biomarkers. Both dosage group (100mg and 40mg) patients had benefits in the bone scan response in 73% and 45%, respectively, as well as reductions in measurable soft tissue disease in 80% and 79%, respectively. However, because cabozantinib is a TKI suppressing both tumor cells and bone cells, the clinical benefits observed in the phase 2 clinical trials may have been confounding effects of suppressing two compartments (i.e. tumor and stroma) at the same time. Indeed, cabozantinib reduced bone turnover blood serum markers such as alkaline phosphatase (ALP, a bone formation marker) and c-telopeptide (CTx, a bone resorption marker), within 12 weeks, indicating that cabozantinib affects the stromal compartment of the tumor microenvironment [34]. Accordingly, to dissect the net effect of c-Met suppression in the stromal compartment alone, we performed preclinical studies using cabozantinib-resistant bone metastatic prostate tumor cells, as well as in vitro studies using c-Met knockdown osteoblasts, and found that the suppression of c-Met specifically in osteoblasts suppressed osteoclastogenesis, tumor-induced osteolysis, and tumor growth in bone [20]. In parallel with our data, Tsai et al. demonstrated that HGF increased bone morphogenetic protein (BMP)-2 in human osteoblasts via c-Met, FAK, JNK, RUNX2, and p300 pathways [35], and Chen et al. demonstrated that HGF increased osteopontin in human osteoblasts via PI3K/AKT, c-Src, and AP-1 pathways [36]. These data suggest that the activation of osteoblasts in the metastatic bone microenvironment is dependent on the growth factors that can stimulate c-Met pathways. To more directly support this idea, Dai et al. showed that cabozantinib has direct anti-tumoral activity in their pre-clinical in vivo mouse models of metastatic prostate cancer, and more importantly, the data suggest that cabozantinib modulates osteoblast activity, which contributes to anti-tumoral efficacy [32,37]. Although the majority of pre-clinical and clinical results are from prostate cancer, c-Met inhibitors have been tested on other bone metastatic cancers, such as breast cancer, and showed a similar clinically efficacy [38]. Watanabe et al. used another c-Met/VEGFR2 dual kinase inhibitor (TAS-115) and showed that the novel inhibitor attenuates FMS-dependent osteoclast differentiation and prostate cancer-induced osteolysis [31,39]. Fioramonti et al. provided additional evidence that cabozantinib decreased tumor-induced osteolysis via direct effects on osteoclasts, as well as indirect effects on osteoblasts (reduction of RANKL and OPG expression), in agreement with our data [40]. Patnaik et al. demonstrated that cabozantinib induced CXCL12 and high mobility group box 1 (HMGB1) proteins, leading to increased neutrophil chemoattraction in the prostate tumor tissues of PTEN/p-53-decifient mice, suggesting that cabozantinib suppresses prostate tumors by activating anti-tumoral innate immunity [41]. Interestingly, short-term cabozantinib treatment (30 mg/kg, 5 × weekly for one or two weeks) in female Balb/c mice suppressed trabecular bone structures and reduced bone marrow cellularity, but increased the number of megakaryocytes. The effects were all transient and disappeared following cessation of the treatment [42]. Collectively, these lines of evidence indicate that HGF/c-Met signaling is important in the microenvironment of bone metastasis, and the efficacy of cabozantinib in bone metastasis is, at least in part, dependent on suppression of the stromal compartment cells, including osteoblasts and osteoclasts. 

## 5. Limitations and Challenges Targeting c-MET in Bone Metastasis

Despite the large amount of data supporting the rationale to target c-Met/HGF in bone metastasis, clinical trial results with c-Met/HGF inhibitors, particularly TKIs, have failed to show favorable results supporting further advancement of the drugs in the clinic. For example, the COMET-1 trial, in which metastatic castration-resistant prostate cancer (CRPC) patients were treated with cabozantinib (60 mg once per day) or prednisone (5 mg twice per day), showed that the cabozantinib group had improved bone biomarkers, bone scan responses (BSR), and radiographic progression-free survival (rPFS), yet failed to satisfy the primary endpoint, i.e., improvement of the overall survival (OS) [43]. In a companion study, the COMET-2 trial, in which progressive mCRPC patients were treated with cabozantinib vs. mitoxantrone (topoisomerase inhibitor)/prednisone, showed no efficacy on the primary endpoint (more than 30% reduction in pain responses), with secondary endpoint (BSR and OS) showing trends favoring cabozantinib treatment. Based on these negative results from two clinical trials with cabozantinib, the c-MET and VEGFR2 dual TKI will not be available for mCRPC patients in the near future. Another c-Met TKI, Tivantinib (also known as ARQ 107), showed no effects in metastatic triple-negative breast cancer, but showed improved PFS in mCRPC patients in Phase 2 clinical trials [44,45]. Tivantinib alone or in combination with zoledronic acid has been shown to suppress breast cancer bone metastasis in an in vivo mouse model [46,47]. However, there are no clinical trials (registered in www.clinicaltrials.gov) ongoing for bone metastasis treated with tivantinib.

In addition to TKIs, there are several monoclonal antibodies blocking c-Met or HGF undergoing clinical development, such as onartuzumab (anti-c-Met antibody), emibetuzumab, LY3164530, JNJ-61186372, rilotuzumab (anti-HGF antibody), ficlatuzumab, etc [48]. These antibodies are currently in various phases of clinical trials or pre-clinical trials, but none of them are being tested for bone metastatic diseases. D’Amico et al. highlighted the roles of cancer stem cells expressing c-Met in renal cancer bone metastasis, and showed that the c-Met inhibitor JNJ-38877605 inhibits osteoclast activation in a preclinical NOD/SCID mouse model [49]. Recently, an interesting Phase 1 trial was registered at the clinicaltrials.gov (NCT01837602) website. The trial tests autologous c-Met redirected T cells administered intra-tumorally in metastatic breast cancer patients. c-Met-targeting chimeric antigen receptor (CAR) T cells showed promising therapeutic efficacy in a pre-clinical model [50], and thus the trial was initiated. 

Even with the negative results from the multiple clinical trials, c-Met/HGF remains an important therapeutic target for metastatic cancers, including bone metastasis. Further extensive research to understand c-Met/HGF regulation in the organ-specific microenvironment is required for the clinical application of novel therapeutics. For example, roles of the extracellular matrix (ECM) and tumor-associated fibroblasts should be considered. Noriega-Guerra et al. described that the ECM is a reservoir for bioactive molecules, including HGF [51]. In addition, ECM components such as proteoglycans, matricelluar proteins, and/or proteases, can negatively regulate c-Met activation by sequestering HGF, and thus it is necessary to consider the effects of ECM components on HGF and c-Met for the development of novel therapeutic strategies. Similarly, Bendinelli et al. demonstrated that HGF and transforming growth factor (TGF)-β, both of which are enriched in the metastatic bone matrix, regulate the mesenchymal-to-epithelial transition (the opposite of the epithelial-to-mesenchymal transition) of bone metastatic breast cancer cells via the suppression of tumor suppressor Wwox (WW domain-containing oxidoreductase) [52,53]. In addition to the ECM, the hypoxic environment in bone metastasis is a key regulator in the network of the biological soluble and structural components of the matrix. In bone metastatic cells under hypoxia, similar patterns of Runx2 and SPARC are observed, with both showing downregulation. Conversely, hypoxia induces Endothelin 1, which upregulates SPARC, and these biological stimuli may be considered prognostic markers of bone metastasis in breast carcinoma patients [54]. Lastly, with the advancement of immuno-oncology, the immune system and tumor immunology warrant consideration. Papaccio et al. have extensively reviewed the relevance of c-Met/HGF in tumor immunity [55]. 

## 6. Conclusions

Since its first discovery as an oncogene, c-Met and its sole ligand HGF have been postulated to play critical roles in tumor progression, particularly tumor-stromal interactions [56,57]. Bone is a unique, complex microenvironment encompassing hard tissue, bone marrow immune cells, loose vasculature, enriched growth factors released from the matrix, and hypoxia. The c-Met and HGF axes play important functions in bone metastasis, and remain promising therapeutic targets for bone metastasis. However, as observed in the results of many clinical trials, the c-Met/HGF signaling is not simple, and the regulation and function of c-Met/HGF in the progression of bone metastasis necessitate extensive further investigation.
